# Efficacy of a short-acting oral fluralaner formulation (WellPet™) against fleas and brown dog ticks in controlled and field conditions

**DOI:** 10.3389/fvets.2026.1771575

**Published:** 2026-03-20

**Authors:** Isabelle Vilela Bonfim, Breno Cayeiro Cruz, Igor Renan Honorato Gatto, Debora Azevedo Borges, Monique Taveira Medeiros, Gessica Ariane de Melo Cruz, Juliana Aparecida do Carmo Emidio Moreira da Silva, Clara Rodrigues Dutra, Carlos Eduardo Marques de Oliveira Filho, Ygor Henrique da Silva, Brena Gava Guimarães, Marcus Antonio Martins Buso, Ferdinando Nielsen de Almeida, Thais Ribeiro Correia, Fabio Barbour Scott, Diefrey Ribeiro Campos

**Affiliations:** 1 Animal Parasitology Department, Clinical Research and Technological Innovation Center in Veterinary Medicine—Laerte Grisi, Veterinary Institute, Federal Rural University of Rio de Janeiro, Seropédica, Brazil; 2 Ourofino Saúde Animal Ltda, São Paulo, Brazil

**Keywords:** acaricide, *Ctenocephalides felis felis*, ectoparasites, flea and tick control, insecticide, isoxazolines, *Rhipicephalus linnaei*, *Rhipicephalus sanguineus* tropical lineage

## Abstract

**Background:**

Fluralaner is an isoxazoline widely used for flea and tick control in dogs, mainly in long-acting formulations. However, the performance of short-acting, lower-dose options remains unexplored in scientific literature. This study evaluated the efficacy of a short-acting oral fluralaner formulation (WellPet™–Ourofino Saúde Animal Ltda, São Paulo, Brazil; 10–22.5 mg of fluralaner per kg of body weight) against *Ctenocephalides felis felis* and *Rhipicephalus sanguineus* sensu lato under controlled and field conditions in southeastern Brazil.

**Methods:**

A randomized, blinded, negative-controlled laboratory study assessed intrinsic efficacy using standardized artificial infestations over 49 days. A randomized, assessor-blinded positive-controlled field trial compared fluralaner with sarolaner in naturally infested dogs living in private households. Fleas and ticks were counted following WAAVP guidelines. Efficacy was calculated using arithmetic means, and statistical analyses were performed with Mann–Whitney and Wilcoxon tests. All analyses were conducted using IBM SPSS Statistics 31.0.

**Results:**

In the controlled study, fluralaner produced a rapid reduction in parasite burdens, achieving 100% efficacy for fleas and ticks as early as Day +2. Efficacy remained between 98.3 and 100% for ticks and 99.7 and 100% for fleas throughout the entire period of evaluation. In the field trial (*n* = 159), both fluralaner and sarolaner markedly reduced parasite loads from Day +7 onward. Efficacy remained above 97% for both parasites in both groups, with no significant differences between treatments at any time point. No product-related adverse events were observed, and the product remained effective even in a region dominated by the tropical lineage of *R. sanguineus* s.l., recently redescribed as *Rhipicephalus linnaei*.

**Conclusion:**

This novel short-acting oral fluralaner formulation demonstrated rapid onset and sustained efficacy under experimental and real-world conditions, supporting its use as an effective option for flea and tick control in areas with high parasite pressure, with an unique periodicity of readministrations every 49 days.

## Highlights

- High efficacy was maintained for at least 49 days under controlled conditions
- Field trial confirmed sustained parasite reduction in naturally infested dogs
- Comparable efficacy to already established isoxazolines under real-world conditions.

## 1 Background

Ticks and fleas are among the most clinically and epidemiologically relevant ectoparasites of dogs, capable of causing dermatological irritation, allergic reactions, anemia, and transmitting several vector-borne pathogens of veterinary and public health importance (1, 2). In Brazil, *Rhipicephalus sanguineus* sensu lato is the tick species most frequently found parasitizing dogs in urban areas, where its markedly endophilic behavior, and ability to complete multiple generations indoors, contribute to persistent infestations (3, 4). Amongst fleas, *Ctenocephalides felis felis* is the most widespread species globally, supported by high reproductive capacity, environmental resilience, and substantial reinfestation potential (5, 6).

Isoxazoline ectoparasiticides have become essential tools for parasite control in small-animal medicine, due to their broad spectrum of activity, rapid onset of action, and sustained efficacy (7). Among them, fluralaner is one of the most extensively studied molecules, with data regarding long-acting formulations for oral administration (25–56 mg/kg) widely available in scientific literature, (8, 9) as well as for its topical application (40 mg/kg) (10, 11), and prolonged-release injectable suspension (15 mg/kg) (12). Published data shows that these formulations provide continuous protection for up to 12 weeks for oral and topical products (8–11), and up to 12 months for the injectable formulation (12). Beyond these extensively documented formulations, other fluralaner-containing products, such as Bravecto 1-month™ (MSD Animal Health), are registered and available on the global market, using lower doses and having shorter durations of action. Additionally, environmental studies conducted in kennel facilities have demonstrated substantial suppression of off-host *R. sanguineus* life stages (13, 14).

For the purposes of the present study, the term short-acting will be used to designate fluralaner formulations whose therapeutic indication involves treatment intervals of less than 12 weeks. Conversely, the term long-acting will be used to refer to presentations of the drug whose duration of action exceeds 12 weeks. Although long-acting ectoparasiticides offer clear benefits for controlling fleas and ticks and preventing vector-borne diseases (15, 16), they are not suitable for all situations. Short-acting ectoparasiticides are indicated for animals exposed to different levels of parasitic challenge, as their shorter duration of action allows greater flexibility of use. They may be employed intermittently in low-risk situations or more frequently in environments with high parasitic pressure, including for rapid, temporary disinfestation prior to travel or short-term exposure (17, 18). Their shorter half-life and lower dosage may reduce the probability and severity of eventual adverse effects (16, 19, 20). In addition, short-acting formulations can be incorporated into rotational control programs, which may lessen selection pressure and help delay the development of resistance in ectoparasite populations (21, 22).

To the best of our knowledge, no peer-reviewed studies have evaluated short-acting fluralaner formulations. In literature, their use has been described for the treatment of generalized demodicosis (23) and *Tunga penetrans* infestations (24), whereas studies involving *C. felis* and *R. sanguineus* l.s. have exclusively addressed long-acting products (8, 10–14), creating a relevant gap in knowledge regarding the performance of shorter-duration formulations under both experimental conditions and natural infestations. This gap is particularly important in Brazil, where intense environmental pressure from *C. felis felis* and *R. sanguineus* s.l. contributes to persistent ectoparasite challenges in urban settings (25, 26).

Considering the epidemiological relevance of these ectoparasites and the operational need for more adaptable parasite-control programs, evaluating short-acting fluralaner formulations becomes clinically meaningful. Therefore, the aim of the present study was to assess the efficacy of a short-acting oral fluralaner formulation (WellPet™–Ourofino Saúde Animal Ltda.; 10–22.5 mg of fluralaner per kg of body weight) for the control of *R. sanguineus* s.l. and *C. felis felis* in dogs, using a combined approach consisting of a controlled laboratory trial and a multicenter field study conducted in southeastern Brazil.

## 2 Methods

### 2.1 Ethical considerations and study approval

*Rhipicephalus sanguineus* s.l. and *C. felis felis* colonies used for the experimental infestations were maintained under authorizations issued by the Animal Ethics Committee of the Veterinary Institute of the Federal Rural University of Rio de Janeiro, under protocols 9812271021 and 4313110419, respectively. The controlled study and the field study were approved by the same institution, under protocols 2886011223 and 7865061223, respectively. The owners responsible for the dogs included in the field study signed an Informed Consent Form, authorizing the participation of their animals in the research.

The study was conducted in accordance with VICH GL9—Good Clinical Practice (27), as well as Animal Research: reporting of *in vivo* Experiments (ARRIVE) (28), and followed the WAAVP guidelines for the evaluation of the efficacy of parasiticides against fleas and ticks on dogs and cats (29, 30).

### 2.2 Experimental animals

#### 2.2.1 Dogs included in the controlled study

Twenty Beagle dogs were selected from the experimental kennel of the Clinical Research and Technological Innovation Center in Veterinary Medicine—Laerte Grisi. The group consisted of 10 males and 10 females, aged 16 to 89 months and weighing 8.6 to 13.25 kg, with no history of ectoparasiticide treatment in the two months preceding the study. During the experiment, the dogs were housed individually in kennels measuring 4.46 m × 1.60 m × 2.0 m, with masonry floors and plastic slated platforms. The animals were fed a super-premium commercial diet twice daily, according to the manufacturer's recommendations, and had continuous access to potable water.

#### 2.2.2 Dogs included in the field study

The field study was conducted in the metropolitan region of Rio de Janeiro, southeastern Brazil. A total of 159 dogs were included, with no restrictions regarding breed or coat type; 88 of which were males and 71 females, ranging in age from 3 to 192 months and weighing between 2 and 52 kg. All animals had been free of ectoparasiticide treatment for at least 90 days before study enrollment. In households with more than one dog, only a single animal was selected for participation, chosen based on the highest pre-treatment flea and/or tick count; the remaining resident dogs were treated concomitantly to prevent reinfestation and standardize the environmental parasite challenge. Throughout the study, all animals were maintained under their owners' usual housing, feeding, and water-provision conditions.

### 2.3 Experimental design: controlled laboratory study

The controlled test was a randomized, negative-controlled, parallel-group laboratory study evaluating the efficacy of an oral fluralaner (WellPet™–Ourofino Saúde Animal Ltda.; 10–22.5 mg of fluralaner per kg of body weight) formulation against *C. felis felis* and *R. sanguineus* s.l.

#### 2.3.1 Parasite colonies

In this study, 50 pairs of *C. felis felis* fleas and 25 pairs of *R. sanguineus* s.l. (tropical lineage) ticks, all unfed and 14 days of age, were used. Flea colonies were maintained on cats and tick colonies on rabbits, under controlled environmental conditions (27 ± 2 °C and 70%−80% relative humidity) at the Clinical Research and Technological Innovation Center in Veterinary Medicine–Laerte Grisi.

#### 2.3.2 Artificial infestations

Dog acclimation occurred on Day −14 (14 days before treatment). Initial infestations were performed on Day −9 with ticks and on Day −8 with fleas. To ensure a uniform parasite challenge at the time of treatment, dogs were reinfested on Day −2 with ticks and on Day −1 with fleas. After treatment (which happened on experimental Day 0), controlled reinfestations were carried out throughout the study: with ticks on days +5, +12, +19, +26, +33, +40, and +47, and with fleas on days +6, +13, +20, +27, +34, +41, and +48.

#### 2.3.3 Randomization and treatment administration

On Day −7, dogs were initially evaluated for parasite retention. Only animals retaining ≥ 20% live attached ticks and ≥ 25% live fleas were considered eligible for the study. Eligible dogs were then ranked in a descending order, according to their live tick counts; in cases of identical tick counts, live flea counts were used as the tiebreaker. Random allocation into two groups (Control and Treated), with eight animals each, was subsequently performed using this ranking to ensure balanced pre-treatment parasite burdens. The four dogs with the lowest parasite counts on Day −7 were excluded prior to randomization.

To maintain the integrity of the study and avoid assessment bias, the trial was conducted under blinded conditions. The individual responsible for administering treatments on Day 0 was not involved in any subsequent procedures, including parasite counting, clinical monitoring, or data collection throughout the experimental period.

On Day −2, all dogs were weighed to confirm the appropriate dosing range. On Day 0, dogs in the treated group received a single oral dose of fluralaner (WellPet™–Ourofino Saúde Animal Ltda.) at 10–22.5 mg/kg, according to manufacturer recommendations (administer one palatable tablet orally, which should not be split, in a single dose, equivalent to 10–22.5 mg of fluralaner per kg of body weight, during or after feeding; the presentation that indicates the recommended dose for each weight range should be used). This range corresponds to the manufacturer's commercial tablet weight categories, ensuring administration of at least the minimum recommended dose. The control group did not receive any antiparasitic treatment during the study. All animals were monitored after administration for potential adverse reactions.

#### 2.3.4 Parasite counting and evaluation

Parasite counts were performed on Days +2, +7, +14, +21, +28, +35, +42, and +49. Fleas were removed and counted using a standardized comb test, with thorough combing of the entire body using a fine-toothed flea comb. Ticks were removed manually by systematic inspection of the entire body surface. All collected parasites were immediately classified as alive or dead; for ticks, attachment status (attached or unattached) was also recorded.

### 2.4 Experimental design: field study

The field study was a randomized, positive-controlled, assessor-blinded field trial evaluating the efficacy of oral fluralaner (WellPet™–Ourofino Saúde Animal Ltda.) against *C. felis felis* and *R. sanguineus* s.l. Households included indoor-only, indoor–outdoor, and outdoor-access environments typical of the metropolitan region, reflecting real-world parasite exposure.

#### 2.4.1 Study location

The field study was conducted in the metropolitan region of Rio de Janeiro, southeastern Brazil, in privately owned households previously selected and authorized for participation.

#### 2.4.2 Inclusion and exclusion criteria

On Day −7, an initial screening was performed with the objective of enrolling at least 50 dogs per parasite/group. On this day, all animals were examined for the presence of live fleas and live attached ticks. Morphological identification of flea (*C. felis felis*) and tick (*R. sanguineus* s.l.) species was confirmed according to the taxonomic keys (31, 32), ensuring accurate parasite classification during the screening process.

In households with more than one eligible dog, only one animal was included in the efficacy analysis, selected based on the highest mean flea and/or tick counts (highest challenge) obtained during the baseline assessments (Days −7 and −2). All other resident dogs, although not included in the efficacy evaluation, were treated concomitantly with the same product assigned to their household group, to eliminate potential reinfestation sources and to standardize environmental parasite pressure.

On Day −2, all dogs were reassessed for quantification of live fleas and ticks and were weighed. To be considered eligible, dogs were required to present, based on Days −7 and −2, a minimum mean of 10 live fleas and/or five live attached ticks. Dogs that did not meet these criteria, or whose owners did not consent to participation, were excluded.

#### 2.4.3 Baseline assessments and randomization

After eligibility confirmation, dogs were allocated, according to the order of household inclusion, into two experimental groups containing at least 50 animals each: a Treated Group, which received oral fluralaner (WellPet™–Ourofino Saúde Animal Ltda.), and a Positive Control Group, which received oral sarolaner (Simparic^®^ Zoetis, Brazil). The use of sarolaner as the positive control was based on the fact that other available fluralaner formulations would not provide adequate comparisons due to different periods of action. Sarolaner, though, has an indicated shorter administration regimen, evidenced from controlled and field studies as demonstrating adequate efficacy against *C. felis* and *R. sanguineus* s.l. This allocation ensured balanced pre-treatment parasite burdens between groups. The study was conducted under blinded conditions: individuals responsible for administering treatments were distinct from those performing parasite counts, and all assessors remained unaware of treatment allocation throughout the study.

#### 2.4.4 Study treatments

On Day 0, dogs in the Treated Group received a single oral dose of fluralaner (WellPet™–Ourofino Saúde Animal Ltda.) at 10–22.5 mg/kg, according to body-weight category. Treatments we conduced following manufacturer's recommendations (administer one palatable tablet orally, which should not be split, in a single dose, equivalent to 10–22.5 mg of fluralaner per kg of body weight, during or after feeding; the presentation that indicates the recommended dose for each weight range should be used). Dogs in the Positive Control Group received a single oral dose of sarolaner (Simparic^®^ Zoetis) at the minimum recommended dose of 2 mg/kg, also respecting manufacturer's recommendations. All dogs were observed after dosing for potential adverse reactions.

#### 2.4.5 Follow-up visits and parasite counting

After treatment, dogs were evaluated for the presence of fleas and ticks on Days +7, +14, +21, +28, and +35 through systematic inspection of the entire body surface. Parasite counts followed the exact procedures described for the controlled study, including the standardized comb test for *C. felis felis* and manual removal of *R. sanguineus* s.l. On Day +35, each dog's participation in the study was concluded.

### 2.5 Data analysis

In both studies (controlled and field), the numbers of live fleas and ticks were tabulated in Microsoft Excel^®^, and arithmetic means and standard deviations were calculated. Product efficacy was determined at each evaluation time point following the recommendations of the WAAVP (29), using the formula:

Efficacy (%) = ((M_c–M_t)/M_c) × 100

Where Mc is the arithmetic mean count of live fleas or live attached ticks in the control group, and Mt is the corresponding mean in the treated group.

The distribution of flea and tick count data was initially assessed for each experimental group and at each evaluation time point using the Shapiro–Wilk test, with a significance level of 5% (*p* ≤ 0.05). Most datasets exhibited non-parametric distribution; therefore, non-parametric statistical methods were applied for inferential analyses.

Comparisons between experimental groups (Treated vs. Positive Control and Negative Control) at each evaluation time point were performed using the Mann–Whitney test, appropriate for independent samples. In the field study, intragroup comparisons between baseline (pre-treatment) counts and each post-treatment evaluation were conducted using the Wilcoxon Signed-Rank test for paired samples. All statistical analyses were performed using IBM SPSS Statistics, version 31.0 (IBM Corp., Armonk, NY, USA).

## 3 Results

### 3.1 Controlled laboratory study

The controlled laboratory experiment was conducted under standardized infestations to evaluate the efficacy of fluralaner against *C. felis felis* and *R. sanguineus* s.l..

All dogs enrolled in the controlled experiment completed the study without adverse reactions. Prior to treatment (Day −7), no statistically significant differences were observed between groups for any baseline variable, including flea burden, tick burden, age, sex distribution, and body weight, confirming adequate comparability between groups (Table 1).

**Table 1:** Baseline characteristics of dogs enrolled in the controlled study.

**Variable**	**Control group (*n* = 8)**	**Treated group (*n* = 8)**	***p-value***
Age (months), mean ± SD	31.25 (± 28.13)	38.50 (± 32.45)	0.4898
Body weight (kg), mean ± SD	11.63 (± 11.54)	10.95 (± 10.93)	0.2632
Sex (M/F)	4/4	4/4	1
Pre-treatment flea count, mean ± SD	61.63 ± 29.9	65.75 ± 25.2	1.000
Pre-treatment tick count, mean ± SD	29.63 ± 10.3	30.00 ± 7.6	0.959

**Figure 1:**
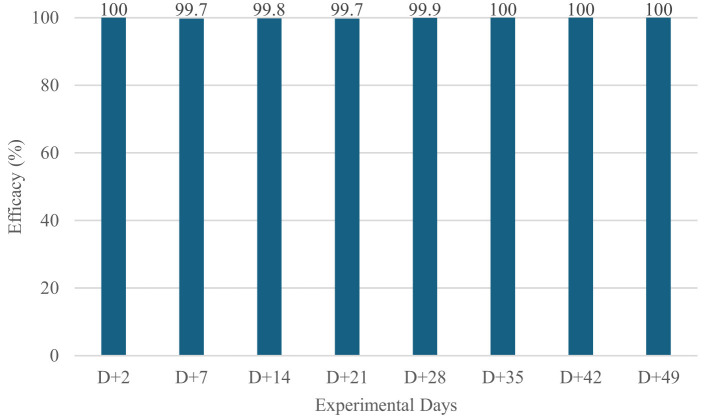
Mean Flea Efficacy of Fluralaner-Treated Dogs (WellPet^®^; Ourofino Saúde Animal) in a Controlled Study.

**Figure 2:**
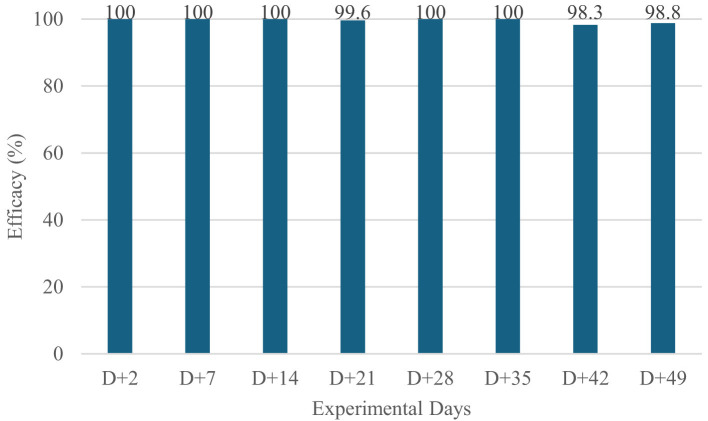
Mean Ticks Efficacy of Fluralaner-Treated Dogs (WellPet^®^; Ourofino Saúde Animal) in a Controlled Study.

Fluralaner induced a rapid and marked reduction in parasite burdens (Table 2, Figures 1, 2). As early as Day +2, both flea and tick counts had decreased, with treated dogs achieving 100% efficacy against both ectoparasites. Throughout the entire post-treatment period (Days +2 to +49), parasite counts in the treated group remained consistently close to zero, and efficacy values ranged from 98.3 to 100% for ticks and 99.7 to 100% for fleas.

**Table 2:** Mean live flea and tick counts and efficacy in untreated and fluralaner-treated (WellPet^®^; Ourofino Saúde Animal) dogs in the controlled study.

**Day**	**Control group (Mean ±SD)**	**Treated group (Mean ±SD)**	**Efficacy (%)**	***p*-value**
**Fleas**	
D + 2	44.75 ± 13.7	0.00 ± 0.0	100.0	< 0.001
D + 7	58.63 ± 19.1	0.25 ± 0.5	99.7	< 0.001
D + 14	76.25 ± 10.6	0.25 ± 0.7	99.8	< 0.001
D + 21	85.25 ± 9.4	0.38 ± 0.7	99.7	< 0.001
D + 28	70.13 ± 11.8	0.13 ± 0.4	99.9	< 0.001
D + 35	80.63 ± 13.4	0.00 ± 0.0	100.0	< 0.001
D + 42	75.00 ± 12.6	0.00 ± 0.0	100.0	< 0.001
D + 49	74.13 ± 14.0	0.00 ± 0.0	100.0	< 0.001
**Ticks**	
D + 2	29.00 ± 12.1	0.00 ± 0.0	100.0	< 0.001
D + 7	26.50 ± 7.3	0.00 ± 0.0	100.0	< 0.001
D + 14	33.75 ± 8.3	0.00 ± 0.0	100.0	< 0.001
D + 21	34.88 ± 3.3	0.25 ± 0.7	99.6	< 0.001
D + 28	35.00 ± 8.5	0.00 ± 0.0	100.0	< 0.001
D + 35	35.63 ± 9.1	0.00 ± 0.0	100.0	< 0.001
D + 42	36.75 ± 8.8	1.00 ± 1.7	98.3	< 0.001
D + 49	35.25 ± 6.9	0.63 ± 0.9	98.8	< 0.001

In contrast, untreated dogs maintained high and stable infestations at all evaluation points. On each post-treatment day, the differences between groups were statistically significant (*p* < 0.001), confirming the efficacy and sustained activity of orally administered fluralaner under standardized and controlled reinfestations.

These results demonstrate that fluralaner provides fast-acting, highly consistent intrinsic efficacy for up to 49 days when ectoparasite challenge is uniform and artificially maintained under laboratory conditions.

### 3.2 Field study

The field study assessed the performance of fluralaner under natural infestation pressures in privately owned dogs living in urban households. A total of 159 naturally infested dogs were included in the field trial, in order to guarantee, in each group, a minimum of 50 animals adequately infested with each target ectoparasite. Baseline characteristics, including parasite burdens, sex, age range, body weight, and coat type, did not differ significantly between the fluralaner-treated group and the positive control group, confirming that the groups were comparable at the start of the study (Table 3). Importantly, no treatment-related adverse events were detected, and owner compliance was high throughout the study.

**Table 3:** Baseline characteristics of dogs enrolled in field study.

**Variable**	**Control group (*n* = 79)**	**Treated group (*n* = 80)**	***p-value***
Age (months), mean ± SD	43.11 (± 34.47)	42.61 (± 41.05)	0.896
Body weight (kg), mean ± SD	15.53 (± 7.71)	16.16 (± 9.11)	0.797
Sex (M/F)	35/45	43/36	0.2347
Breed (pure/mixed)	14/66	22/57	0.1190
Coat length (short/medium/long)	66/11/3	63/14/2	0.7322
Presence of parasites (flea/ticks/flea + ticks)	22/28/30	25/26/28	0.8503
Pre-treatment flea count, mean ± SD^*^	28.47 (± 26.58)	30.69 (± 53.95)	0.722
Pre-treatment tick count, mean ± SD^*^	20.13 (± 41.74)	16.61 (± 10.77)	0.662

^*^Pre-treatment parasite counts represent the arithmetic mean of the counts obtained on Days −7 and −2.

**Figure 3:**
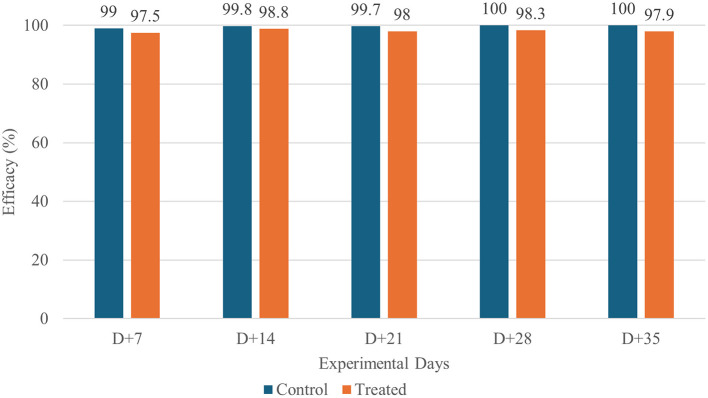
Flea Efficacy in Dogs Treated with Fluralaner (WellPet™) and Sarolaner in a Field Study.

**Figure 4:**
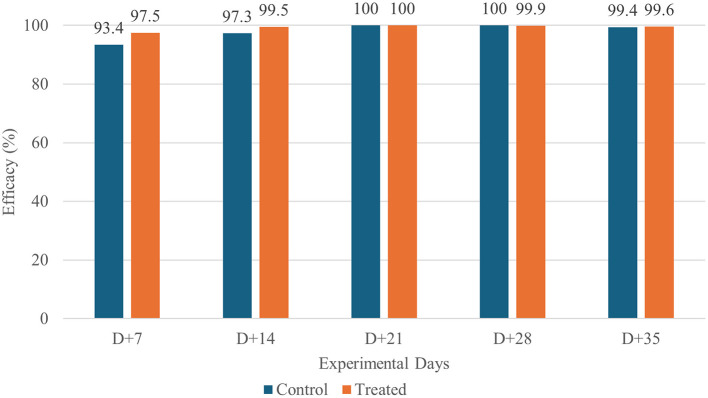
Tick Efficacy in Dogs Treated with Fluralaner (WellPet™) and Sarolaner in a Field Study.

Under natural environmental exposure, both treatments produced substantial reductions in flea and tick burdens. By Day +7, mean counts had already declined sharply in both groups, and parasite burdens continued to decrease throughout the observation period. Efficacy remained high (> 97%) in both groups across all evaluation days (Table 4, Figures 3, 4).

**Table 4:** Mean live flea and tick counts and efficacy in positive-control (Simparic^®^; Zoetis) and fluralaner-treated (WellPet™–Ourofino Saúde Animal Ltda.) dogs in the field study.

**Day**	**Group**	**Mean (±SD)**	**Efficacy (%)**	***p*-value (intragroup)**	***p*-value (intergroup)**
**Fleas**	
Pretreatment^*^	Control	28.47 (± 26.58)	—	—	0.722
	Treated	30.69 (± 53.95)	—	—	
D + 7	Control	0.29 (± 0.89)	98.99	< 0.0001	0.99
	Treated	0.75 (± 2.37)	97.54	< 0.0001	
D + 14	Control	0.06 (± 0.24)	99.8	< 0.0001	0.267
	Treated	0.36 (± 1.09)	98.83	< 0.0001	
D + 21	Control	0.08 (± 0.33)	99.73	< 0.0001	0.673
	Treated	0.60 (± 2.44)	98.03	< 0.0001	
D + 28	Control	0.00 (± 0.00)	100	< 0.0001	0.024
	Treated	0.53 (± 1.86)	98.28	< 0.0001	
D + 35	Control	0.00 (± 0.00)	100	< 0.0001	0.024
	Treated	0.66 (± 2.48)	97.85	< 0.0001	
**Ticks**	
Pretreatment^*^	Control	30.13 (± 41.74)	—	—	0.662
	Treated	15.61 (± 10.77)	—	—	
D + 7	Control	1.33 (± 6.59)	93.4	< 0.0001	0.418
	Treated	0.39 (± 1.11)	97.51	< 0.0001	
D + 14	Control	0.53 (± 3.19)	97.34	< 0.0001	0.199
	Treated	0.06 (± 0.41)	99.54	< 0.0001	
D + 21	Control	0.00 (± 0.00)	100	< 0.0001	1
	Treated	0.00 (± 0.00)	100	< 0.0001	
D + 28	Control	0.00 (± 0.00)	100	< 0.0001	0.3
	Treated	0.02 (± 0.14)	99.88	< 0.0001	
D + 35	Control	0.12 (± 0.92)	99.4	< 0.0001	0.532
	Treated	0.06 (± 0.30)	99.64	< 0.0001	

^*^Pre-treatment parasite counts represent the arithmetic mean of the counts obtained on Days –7 and –2.

No statistically significant differences were observed between fluralaner-treated group and positive control group at any time point, indicating comparable performance between the two orally administered isoxazolines under real-world conditions.

## 4 Discussion

Although the efficacy of fluralaner has already been demonstrated for oral, topical, and injectable formulations against fleas and ticks (8–12), the present study evaluates a novel, lower-dose commercial formulation (10–22.5 mg/kg) designed to provide shorter protection of up to 49 days for *C. felis felis* and *R. sanguineus* s.l. control. This approach addresses an important clinical need, as shorter dosing intervals may offer increased flexibility for veterinarians, improve integration with monthly parasite-control programs, and potentially reduce prolonged selective pressure on ectoparasite populations (16, 21, 22). The shorter duration of action is related to the fact that the short-acting formulation uses a lower dose than that employed in treatment with long-acting oral fluralaner.

Shorter readministration periods tend to maintain more stable and consistently elevated plasma concentrations, as reapplication occurs while the drug is still present at therapeutic levels in the organism. In contrast, long-acting formulations or higher-dose extended-interval regimens exhibit a gradual decline in systemic concentration over time, and by the moment of re-dosing, plasma levels may fall below the optimal threshold, potentially resulting in reduced clinical efficacy. Consequently, reduced dosing schedules may provide greater pharmacodynamic consistency and minimize the risk of subtherapeutic windows (33, 34).

Moreover, short-duration formulations containing reduced doses of fluralaner may offer specific advantages in situations where lower systemic persistence is desirable. Products with shorter pharmacological exposure inherently limit the duration of active compound circulation within the organism, which may be beneficial in cases of suspected adverse events or when rapid therapeutic adjustments are required (17, 19, 20). This pharmacological profile provides veterinarians with greater flexibility to individualize ectoparasite control strategies, particularly for dogs with comorbidities, a history of drug sensitivity or those undergoing concurrent treatments. By reducing long-term systemic exposure without compromising early efficacy, lower-dose fluralaner formulations expand the range of safe and practical options available for the clinical management of flea and tick infestations.

Fluralaner has a well-established safety profile in dogs, with demonstrated tolerability across oral, topical and injectable formulations, even at doses substantially higher than those used in the present study (35–37). Although a formal safety assessment was not an explicit objective of this investigation, no adverse events were observed in any of the dogs enrolled in either the controlled or the field study. These findings are consistent with the extensive literature supporting the safety of fluralaner and align with the broad therapeutic margin described for this compound.

Controlled studies evaluating different fluralaner formulations have demonstrated similar levels of efficacy against *C. felis* and *R. sanguineus*. After 49 days of treatment, the oral formulation (25–56 mg/kg) showed efficacy ranging from 95 to 100% (8, 9), while the topical formulation ranged from 98.3 to 100% (10, 11) and the injectable from 99 to 100% (10). In the controlled study conducted here, the fluralaner-treated group maintained comparable efficacy values, remaining between 98.3 and 100% throughout the 49-day assessment period, confirming the consistent performance of the active ingredients regardless of the route of administration.

Unlike the controlled study, the field trial displayed substantial heterogeneity within the experimental group, including variations in age, weight, hair coat length, and household types. This diversity is intentional and essential to confirm the performance of an ectoparasiticide under real infestation conditions (29). Despite this heterogeneity, the fluralaner-treated group maintained high efficacy, ranging from 93.4 to 100% throughout the 35 days following treatment. Nevertheless, both treatment groups stabilized at low parasite counts and maintained high levels of control across all visits.

The initial efficacies observed in the field study were lower than those in the controlled study, a result of the constant reinfestation that occurs during the first days, when environmental infestation levels are still high (13, 14, 38). However, as the study progresses and reinfestation pressure decreases, efficacy increases and approaches the values obtained in the controlled setting. This pattern demonstrates that, even with a lower dose and a shorter recommended treatment period, the evaluated formulation is effective in promoting the control of environmental infestations.

Furthermore, it is important to note that in southeastern Brazil the tropical lineage of *R. sanguineus* s.l. is the predominant variant, as documented in epidemiological studies (39–41). This lineage, recently reclassified as *R. linnaei*, is highly adapted to warm climates and tends to maintain stable populations throughout the year, thereby increasing infestation pressure on dogs living in urban and peri-urban environments. The efficacy observed in the present study demonstrates that the evaluated formulation can act consistently even against this lineage, supporting its potential to control different populations of *R. sanguineus* s.l. occurring in the country.

This study presents several limitations that should be considered. The follow-up period, restricted to a maximum of 49 days, prevents conclusions about the exact point at which efficacy begins to decline or how the product performs over longer intervals. In addition, no formal assessment of pharmacokinetic or safety parameters was conducted (in this study, as these factors were investigated in different trials, whose data is yet unpublished), which could have contributed to a better understanding of the systemic behavior of this lower-dose formulation compared to traditional long-acting presentations. Future research should prioritize characterizing and investigating the performance of this short duration formulation under different environmental conditions, including regions with varying climates and distinct lineages of *R. sanguineus* s.l. Assessing monthly re-administration schedules and integrating this formulation into broader parasite-control programs may further strengthen the evidence supporting its clinical applicability.

## 5 Conclusion

This study shows that the short-acting oral fluralaner (WellPet™–Ourofino Saúde Animal Ltda.) formulation provides sustained control of *C. felis felis* and *R. sanguineus* s.l. under both controlled and field conditions. These findings support its use as an effective monthly option for flea and tick control in regions with high parasite pressure.

## Data Availability

The raw data supporting the conclusions of this article will be made available by the authors, without undue reservation.

## References

[1] Pereira A Cruz A Novo T Maia C. *Ctenocephalides felis* (cat flea). Trends Parasitol. (2025) 41:249–50. doi: 10.1016/j.pt.2024.12.016 39818462

[2] Dantas-Torres F de Sousa-Paula LC Otranto D. The *Rhipicephalus sanguineus* group: updated list of species, geographical distribution, and vector competence. Parasit Vectors. (2024) 17:540. doi: 10.1186/s13071-024-06572-3 39731169 PMC11681662

[3] Novelo M Clack W Siefker M Brodigan J Rubino F Foley J . Rhipicephalus sanguineus sensu lato infestation in an urban area in South Sacramento, California, USA. Parasit Vectors. (2025) 18:430. doi: 10.1186/s13071-025-07069-3 41146265 PMC12560577

[4] Jongejan F Uilenberg G. The global importance of ticks. Parasitology. (2004) 129:S3–S14. doi: 10.1017/S0031182004005967 15938502

[5] Rust M. The Biology and ecology of cat fleas and advancements in their pest management: a Review. Insects. (2017) 8:118. doi: 10.3390/insects8040118 29077073 PMC5746801

[6] Moore CO André MR Šlapeta J Breitschwerdt EB. Vector biology of the cat flea *Ctenocephalides felis*. Trends Parasitol. (2024) 40:324–37. doi: 10.1016/j.pt.2024.02.006 38458883 PMC11168582

[7] Zhou X Hohman AE Hsu WH. Current review of isoxazoline ectoparasiticides used in veterinary medicine. J Vet Pharmacol Ther. (2022) 45:1–15. doi: 10.1111/jvp.12959 33733534

[8] Taenzler J Wengenmayer C Williams H Fourie J Zschiesche E Roepke RK . Onset of activity of fluralaner (BRAVECTOTM) against *Ctenocephalides felis* on dogs. Parasit Vectors. (2014) 7:567. doi: 10.1186/PREACCEPT-1563099569137345 25471474 PMC4263043

[9] Beugnet F Liebenberg J Halos L. Comparative efficacy of two oral treatments for dogs containing either afoxolaner or fluralaner against *Rhipicephalus sanguineus* sensu lato and *Dermacentor reticulatus*. Vet Parasitol. (2015) 209:142–5. doi: 10.1016/j.vetpar.2015.02.002 25716658

[10] Taenzler J Liebenberg J Mienie M Everett WR Young DR Vihtelic TS . Efficacy of fluralaner spot-on solution against induced infestations with *Rhipicephalus sanguineus* on dogs. Parasit Vectors. (2016) 9:276. doi: 10.1186/s13071-016-1523-4 27241176 PMC4886405

[11] Gopinath D Meyer L Smith J Armstrong R. Topical or oral fluralaner efficacy against flea (*Ctenocephalides felis*) transmission of *Dipylidium caninum* infection to dogs. Parasit Vectors. (2018) 11:557. doi: 10.1186/s13071-018-3140-x 30359284 PMC6202868

[12] Raulf M-K Raue K Schwarz A Petersen I Zschiesche E Heinau L . Single treatment with a fluralaner injectable suspension (Bravecto^®^ injectable) provides 1-year efficacy against *Rhipicephalus sanguineus* sensu lato and *Ctenocephalides felis* in dogs. Parasit Vectors. (2024) 17:438. doi: 10.1186/s13071-024-06535-8 39462404 PMC11514862

[13] Allen K Little S Petersen M Gruntmeir J Barrett A Herrin B . Evaluation of oral fluralaner (Bravecto^®^) for efficacy against nymphs of *Amblyomma americanum* and *Rhipicephalus sanguineus* (sensu lato). Parasit Vectors. (2020) 13:315. doi: 10.1186/s13071-020-04179-y 32552774 PMC7302130

[14] Labruna MB Doretto JS de Araújo Nascimento OC Barufi FB Rosa SC Osowski GV . Efficacy of either orally administered fluralaner or topically administered imidacloprid/flumethrin for controlling *Rhipicephalus sanguineus* sensu lato premises infestations. Parasit Vectors. (2023) 16:414. doi: 10.1186/s13071-023-06028-0 37964390 PMC10647063

[15] Joachim A Robertson LJ Ferroglio E Bäumer W Leschnik M. Antiparasitics against ectoparasites in small animals– important pharmaceutical substances or underestimated environmental hazards? Vet Parasitol. (2025) 339:110557. doi: 10.1016/j.vetpar.2025.110557 40752179

[16] Beugnet F Franc M. Insecticide and acaricide molecules and/or combinations to prevent pet infestation by ectoparasites. Trends Parasitol. (2012) 28:267–79. doi: 10.1016/j.pt.2012.04.004 22627116

[17] Little SE. Greene's infectious diseases of the dog and cat. in Fleas and Lice. Amsterdam: Elsevier (2021). p. 1324–37. doi: 10.1016/B978-0-323-50934-3.00106-3

[18] Rust MK Waggoner MM Hinkle NC Stansfield D Barnett S. Efficacy and longevity of nitenpyram against adult cat fleas (Siphonaptera: Pulicidae). J Med Entomol. (2003) 40:678–81. doi: 10.1603/0022-2585-40.5.678 14596282

[19] Rust M. Advances in the control of (cat flea) on cats and dogs. Trends Parasitol. (2005) 21:232–6. doi: 10.1016/j.pt.2005.03.010 15837612

[20] Loke YK Mattishent K. Rang and Dale's Pharmacology, 2nd Edn.Amsterdam: Elsevier (2019).

[21] Coles TB Dryden MW. Insecticide/acaricide resistance in fleas and ticks infesting dogs and cats. Parasit Vectors. (2014) 7:8. doi: 10.1186/1756-3305-7-8 24393426 PMC3891977

[22] Makwarela TG Seoraj-Pillai N Nangammbi TC. Tick control strategies: critical insights into chemical, biological, physical, and integrated approaches for effective hard tick management. Vet Sci. (2025) 12:114. doi: 10.3390/vetsci12020114 40005873 PMC11860501

[23] Rohdich N Meyer L Guerino F. Fluralaner 546% (w/w) flavored chewable tablet (Bravecto^®^ 1-Month) is effective for treatment of canine generalized demodicosis. Parasit Vectors. (2022) 15:83. doi: 10.1186/s13071-022-05213-x 35279216 PMC8917636

[24] dos Santos KC Guedes PEB Albuquerque GR de Jesus AV da Paixão Sevá A de Oliveira JTS . Efficacy of monthly treatment with oral fluralaner (Bravecto^®^ 1-Month) against *Tunga penetrans* in dogs in Brazil: a randomized, double-blind, controlled field study. Parasit Vectors. (2024) 17:197. doi: 10.1186/s13071-024-06272-y 38685048 PMC11059606

[25] Araes-Santos AI Moraes-Filho J Peixoto RM Spolidorio MG Azevedo SS Costa MM . Ectoparasite infestations and canine infection by rickettsiae and ehrlichiae in a semi-arid region of Northeastern Brazil. Vector Borne Zoonotic Dis. (2015) 15:645–51. doi: 10.1089/vbz.2015.1786 26565771 PMC4652196

[26] Dantas-Torres F Otranto D. Dogs, cats, parasites, and humans in Brazil: opening the black box. Parasit Vectors. (2014) 7:22. doi: 10.1186/1756-3305-7-22 24423244 PMC3914713

[27] VICH Expert Working Group. VICH. GL9: Good Clinical Practice. Brussels: VICH Expert Working Group (2000).

[28] Percie du Sert N Hurst V Ahluwalia A Alam S Avey MT Baker M . The ARRIVE guidelines 2.0: updated guidelines for reporting animal research. PLoS Biol. (2020) 18:e3000410. doi: 10.1371/journal.pbio.3000410 32663219 PMC7360023

[29] Marchiondo AA Holdsworth PA Fourie LJ Rugg D. Hellmann K. World association for the advancement of veterinary parasitology (WAAVP) second edition: guidelines for evaluating the efficacy of parasiticides for the treatment, prevention and control of flea and tick infestations on dogs and cats. Vet Parasitol (2013) 194:84–97. doi: 10.1016/j.vetpar.2013.02.003 23741753

[30] Marchiondo AA Holdsworth PA Green P Blagburn BL Jacobs DE. World association for the advancement of veterinary parasitology (WAAVP) guidelines for evaluating the efficacy of parasiticides for the treatment, prevention and control of flea and tick infestation on dogs and cats. Vet Parasitol. (2007) 145:332–44. doi: 10.1016/j.vetpar.2006.10.028 17140735

[31] Wall R Shearer D. Wall & Shearer (2001) and Walker et al. (2003) 2nd Edn. Oxford: Blackwell Science (2001).

[32] Walker AR Bouattour A Camicas J-L Estrada-Peña A Horak IG . Ticks of Domestic Animals in Africa: A Guide to Identification of Species. Edinburgh: Bioscience Reports (2003).

[33] Grover A Benet LZ. Intermittent drug dosing intervals guided by the operational multiple dosing half lives for predictable plasma accumulation and fluctuation. J Pharmacokinet Pharmacodyn. (2011) 38:369–83. doi: 10.1007/s10928-011-9198-0 21499748 PMC3677834

[34] Begg EJ. Clinical pharmacokinetics. In: Instant Clinical Pharmacology. Chichester: Wiley-Blackwell (2008). p. 1–14

[35] Walther FM Allan MJ Roepke RK Nuernberger MC. Safety of fluralaner chewable tablets (BravectoTM), a novel systemic antiparasitic drug, in dogs after oral administration. Parasit Vectors. (2014) 7:87. doi: 10.1186/1756-3305-7-87 24606886 PMC3975339

[36] Petersen I Goebel-Lauth S Pobel T Gil MJ Löhlein W Wolf O . Clinical efficacy and safety of a single administration of fluralaner injectable suspension (BRAVECTO^®^ injectable) vs. monthly administration of oral afoxolaner (NexGard^®^) in dogs for tick and flea control over one year under European field conditions. Parasit Vectors. (2024) 17:504. doi: 10.1186/s13071-024-06590-1 39654069 PMC11626764

[37] Rohdich N Roepke RK Zschiesche E. A randomized, blinded, controlled and multi-centered field study comparing the efficacy and safety of BravectoTM (fluralaner) against FrontlineTM (fipronil) in flea- and tick-infested dogs. Parasit Vectors. (2014) 7:83. 3 doi: 10.1186/1756-3305-7-83 24593931 PMC3975895

[38] Dryden MW. Epidemiology and control of fleas infesting dogs and cats. Vet Q. (1996) 18:44–5. doi: 10.1080/01652176.1996.9694672 22074526

[39] Santos HF Flausino W Martins TF Silito IS Luz HR Serpa MC de A . Ticks (Acari: Ixodidae) and tick-borne agents associated with domestic dogs in an environmental protection area in Brazil, with molecular evidence of *Rhipicephalus linnaei* (Audouin, 1826). Rev Bras Parasitol Vet. (2024) 33:e008224. doi: 10.1590/s1984-29612024045 39383386 PMC11486457

[40] Sanches GS Évora PM Mangold AJ Jittapalapong S Rodriguez-Mallon A Guzmán PEE . Molecular, biological, and morphometric comparisons between different geographical populations of Rhipicephalus sanguineus sensu lato (Acari: Ixodidae). Vet Parasitol. (2016) 215:78–87. doi: 10.1016/j.vetpar.2015.11.007 26790741

[41] Moraes-Filho J Marcili A Nieri-Bastos FA Richtzenhain LJ Labruna MB. Genetic analysis of ticks belonging to the *Rhipicephalus sanguineus* group in Latin America. Acta Trop. (2011) 117:51–5. doi: 10.1016/j.actatropica.2010.09.006 20858451

